# Risk Comparison of Filler Embolism Between Polymethyl Methacrylate (PMMA) and Hyaluronic Acid (HA)

**DOI:** 10.1007/s00266-019-01320-w

**Published:** 2019-03-01

**Authors:** Fangfei Nie, Hongbin Xie, Guanhuier Wang, Yang An

**Affiliations:** Department of Plastic Surgery, Peking University Third Hospital, 49 North Garden Rd., Haidian District, Beijing, 100191 People’s Republic of China

**Keywords:** Polymethyl methacrylate (PMMA), Hyaluronic acid (HA), Intra-arterial injection, Embolism, Necrosis

## Abstract

**Background:**

The incidence of vascular complications varies among different fillers. The main purpose of this study was to compare the risk of embolism between PMMA (Artecoll) and hyaluronic acid (HA, Restylane) after artery injection.

**Methods:**

Rabbit ears were injected via the central artery with 0.1 ml PMMA (group A), 0.1 ml HA (group B), 0.2 ml PMMA (group C), or 0.2 mL HA (group D), respectively. The formation of transparent emboli was monitored right after injection. Tissue necrosis and histopathological changes were analyzed on day 7.

**Results:**

With 0.1 ml injected volume, PMMA was dispersed within a few minutes and only 5% of the injected ears had mild necrosis on day 7, while HA tended to form obvious transparent emboli, an indication of blood vessel clotting, and 60% of injected ears showed necrosis on day 7. With 0.2 ml injected volume, PMMA had a risk of complete blood vessel clotting in between 0.1 ml PMMA group and 0.1 ml HA group, and 30% of injected ears had necrosis; in contrast, 100% of 0.2 ml HA-injected ears showed transparent emboli and necrosis. The necrosis areas were significantly increased in the HA groups compared with PMMA groups at the same injection volumes. HA injection also caused dilation of small blood vessels.

**Conclusion:**

At the same injection volume, PMMA had less risk of embolism compared with HA. With increased injection volume, there were increased risks of embolism and necrosis for both PMMA and HA.

**No Level Assigned:**

This journal requires that authors assign a level of evidence to each article. For a full description of these Evidence-Based Medicine ratings, please refer to the Table of Contents or the online Instructions to Authors www.springer.com/00266.

**Electronic supplementary material:**

The online version of this article (10.1007/s00266-019-01320-w) contains supplementary material, which is available to authorized users.

## Introduction

The minimally invasive injection of cosmetic fillers has been rapidly popularized in the field of cosmetic surgery. It has also brought complications [1]. Hyaluronic acid (HA) is a popular filler. It has been associated with vascular complications and more serious complications, such as blindness or paralysis [2–4]. The main ingredient of HA filler is the viscous sodium hyaluronate gel; therefore, the clinical complications may be related to the high hydrophilicity and high viscosity of HA [5], which make it more difficult to disperse with blood flow. Artecoll is another popular filler but has rarely been associated with tissue necrosis. Artecoll consists of polymethyl methacrylate (PMMA) microspheres (20% by volume, 40 μm in diameter) suspended in 3.5% bovine collagen solution and 0.3% lidocaine [6, 7]. We speculate that low risk of vascular complication associated with Artecoll may be related to small microspheres of PMMA and soluble collagen.

In spite of these clinical observations, there has been no study to compare the potential risks of PMMA and HA in a controlled experimental setting. In this study, the rabbit was used as an animal model to test the hypothesis that different fillers have different risks of blood vessel clotting and tissue necrosis. The central artery of each rabbit ear was injected intra-arterially with one filler at a designated volume to simulate the clinical local injection of filling agent. Dispersion of an injected filler, local tissue necrosis, and local blood vessel diameters were used for risk comparisons between PMMA and HA.

## Materials and Methods

### Animal and Tested Products

Healthy male white rabbits (2.6–3.6 kg) were provided by the experimental animal center at Peking University Health Science Center. The rabbits were housed in metal cages with free access to food and water on natural light at 22 ± 2 °C with 60 ± 10% relative humidity at the experimental animal center at Peking University Health Science Center. The rabbits were killed by intracardiac injection of potassium chloride 7 days after intra-arterial injection of a filler. The tested products include PMMA (Artecoll; Hafod B.V., Rotterdam, the Netherlands) and hyaluronic acid (Restylane; Q-Med, Uppsala, Sweden). All procedures used in this study were approved (No: 2015-0126) by the Experimental Animal Ethics Committee at our institute, and related guidelines for the care and use of animals were followed.

### Animal Model of Intravascular Embolization

Four groups were set up in this experiment: 0.0.1 ml PMMA (group A), 0.1 ml HA (group B), 0.2 ml PMMA (group C), and 0.2 ml HA (group D). The two ears of each rabbit were injected with the same amount of PMMA and HA, respectively. Occasionally, an injection failed in one ear and only one ear of a rabbit was available for injection. *N* = 20 ears for 0.1 ml PMMA and 0.1 ml HA groups; and *N* = 10 ears for 0.2 ml PMMA and 0.2 ml HA groups.

**Fig. 1:**
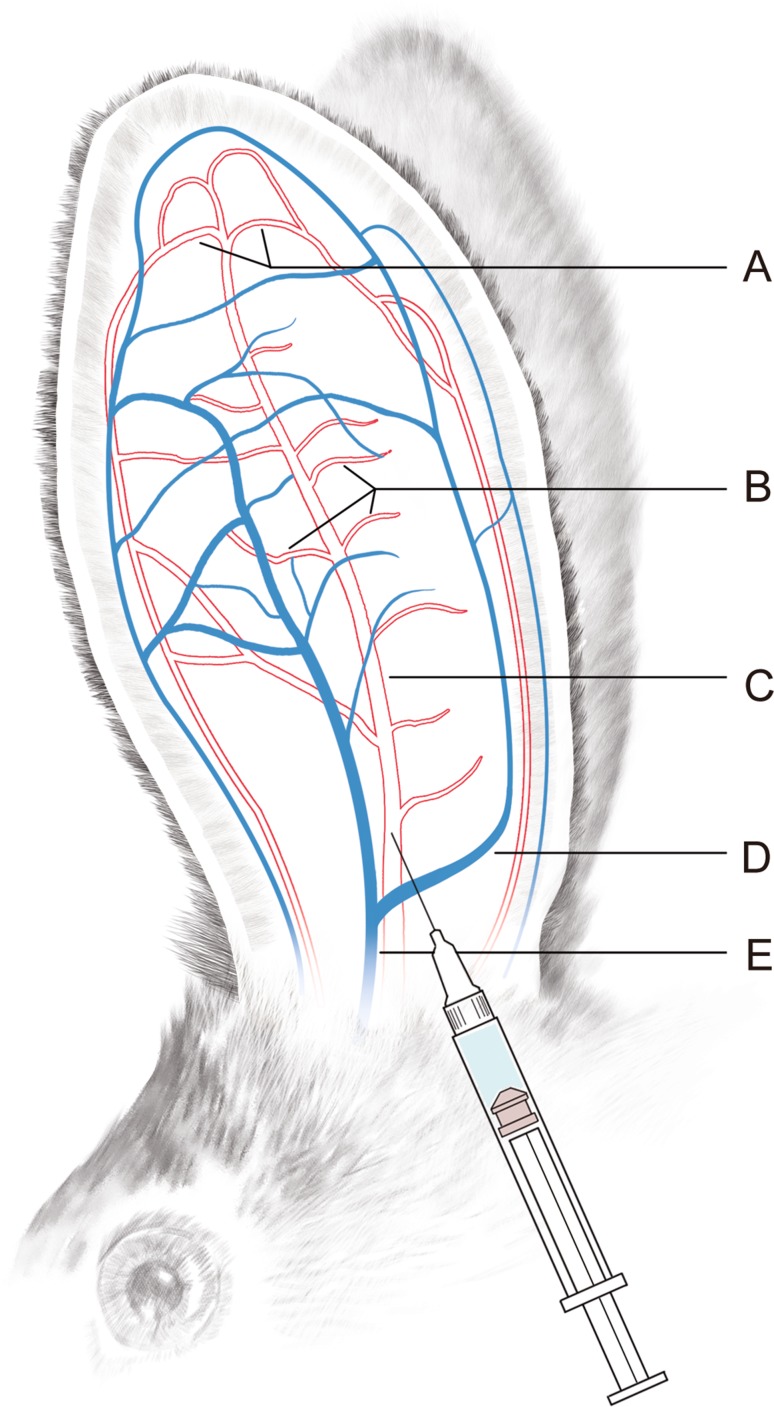
Schematic illustration of the main blood vessels in the rabbit ear and the injection site. A, terminal-end branches of central ear artery (CEA); B, side branches of CEA; C, main trunk of CEA; D, medial ramus of central ear vein (CEV); E, main trunk of CEV. Injection site lies approximately 1 cm above the medial ramus of CEV

The rabbits were fixed in a specially constructed box. The injection site on each ear was shaved and applied with lidocaine cream and cleaned with a saline gauze 30 min later. The ventral side of the rabbit ear was then irradiated with a strong light, and a filler was injected into the main trunk of the central ear artery (CEA) with a 27-G needle (0.5 mm) from 1 cm above the medial ramus of the central ear vein (CEV), which is about 4–5 cm from the root of the rabbit ear [8] (Fig. 1). The speed of injection was controlled to about 0.2 ml/min.

### Observation of CEA Blood Flow and Skin Necrosis

The blood flow was observed and recorded using a camera (Canon 70D with a 24-105 lens; Japan) before injection and within 5 min after injection. Despite a wide range of blood flow conditions after injection of fillers, the main changes of blood flow occurred within 5 min of injection. Therefore, we chose 5 min as the time point for short-term recovery of blood flow. The countercurrent rate was calculated as the percentage of ears with reflux of injected filler passing the injected site. The blood flow condition, skin color, the degree and area of necrosis on each injected rabbit ear were examined and photographed on day 1 and day 7 after injection, respectively. The area of the damaged skin on the ear was quantified using ImageJ software.

### Histological Examination

The proximal, middle, and distal segments of each ear were collected, fixed in 4% formaldehyde, and processed for hematoxylin and eosin (H&E) staining. The longest diameter of the largest vessel in each distal tissue was quantified using ImageJ software.

### Statistical Analysis

Frequency and percentage were analyzed using the Chi-square test or trend Chi-square test. The area of skin lesion and diameter of the largest vessel were analyzed using a nonparametric test. Body weight was analyzed using one-way ANOVA. When applicable, data were expressed as mean ± standard deviation. The significance level for comparisons between two groups was adjusted to 0.0125 according to Bonferroni correction.

## Results

### Immediate Effects of Intra-Arterial Injection of PMMA and HA on Blood Vessels

**Fig. 2:**
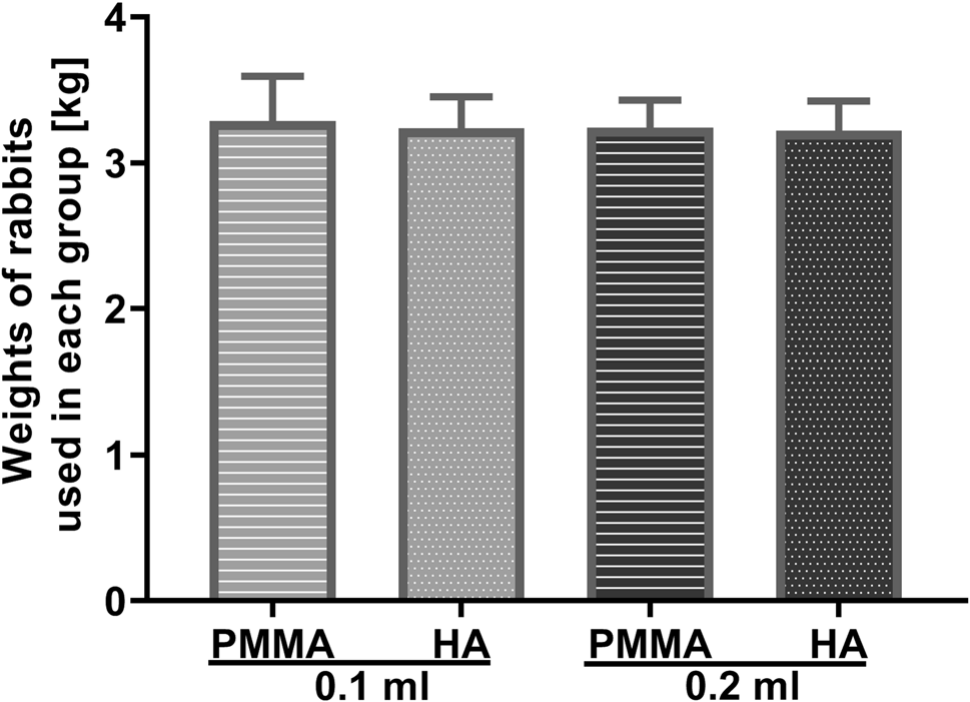
Body weight. *N* = 20 (0.1 ml groups) and 10 (0.2 ml groups); error bar, standard deviation

There was no significant difference in the body weights among the four groups (Fig. 2), which were 3.29 ± 0.31 kg (0.1 ml PMMA, *N* = 20), 3.24 ± 0.22 kg (0.1 ml HA, *N* = 20), 3.24 ± 0.19 kg (0.2 ml PMMA, *N* = 10), and 3.22 ± 0.20 kg (0.2 ml HA, *N* = 10), respectively.

**Fig. 3:**
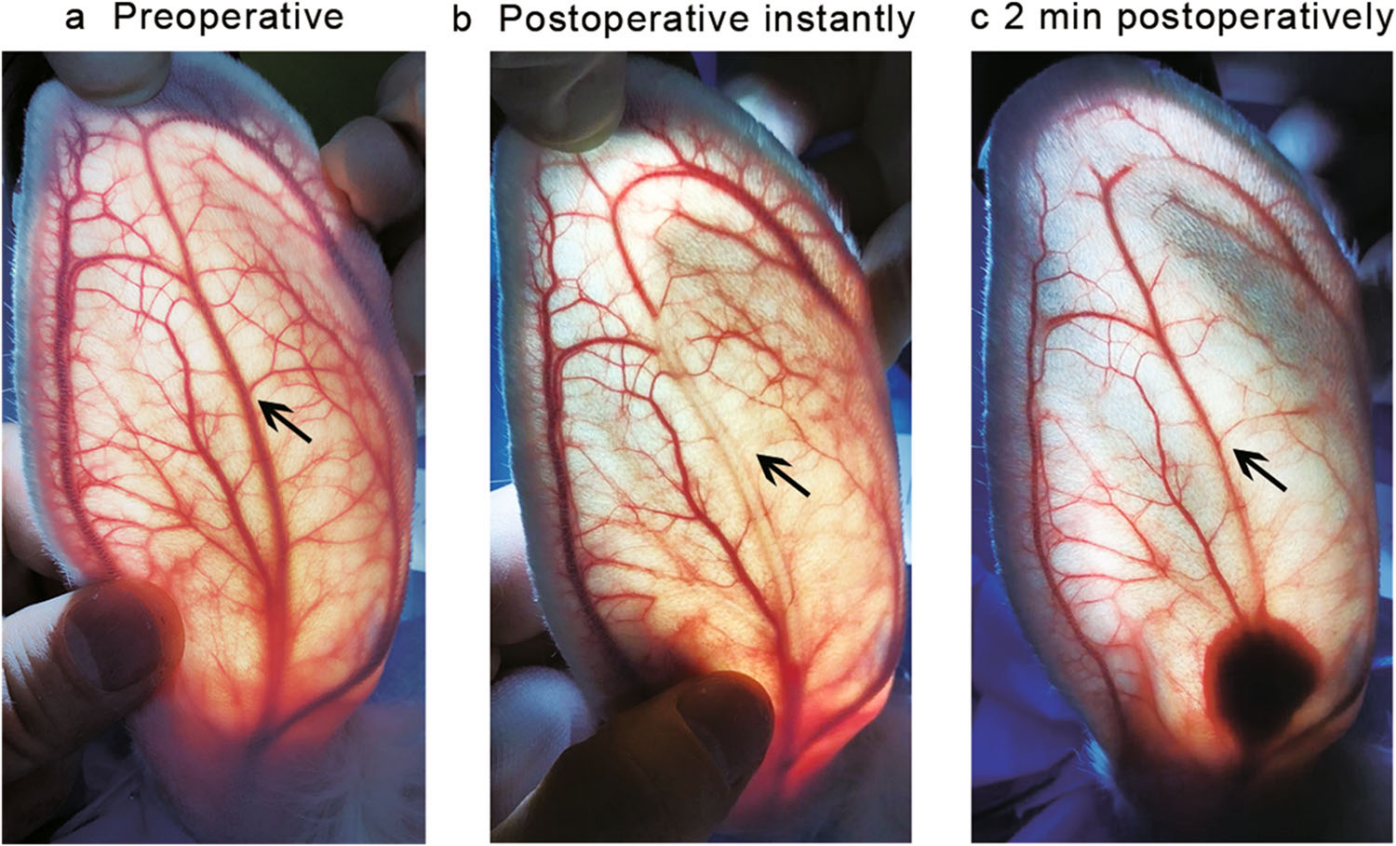
Vascular recanalization immediately after injection of 0.1 ml PMMA. **a** Blood vessels prior to injection. **b** Blood vessels right after injection. Arrow, lack of blood in the main trunk of the central ear artery, indicates the blockage of blood flow. **c** Restoration of blood flow in the main truck 2 min after withdrawal of the needle

**Fig. 4:**
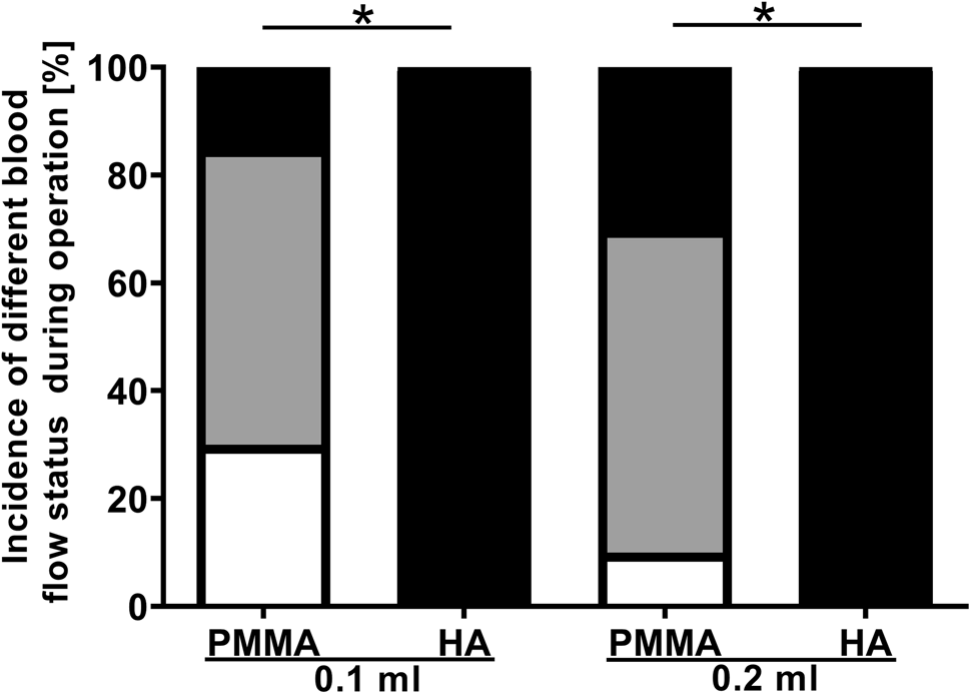
Incidence of different blood flow statuses within 5 min of filler injection: white (I): never blocked; gray (II): temporarily blocked and then restored within 5 min; black (III): sustained blockage of the main branch of central ear artery at 5 min. *p < 0.0125; *N* = 20 (0.1 ml groups) and 10 (0.2 ml groups)

Before injection, the rabbit ear blood vessels were clearly visible under strong light (Fig. 3a). The terminal branches of the central ear artery were Y-shaped, and the main trunk of the central artery and the central vein was easily distinguishable. During injection, the fillers were gradually filling the arteries along the main branch and the side branches of the central ear artery. The short-term responses were grouped into three categories (I, II, III) with I being the mildest and III being the most severe: (I) the fillers were dispersed along the arteries without blood vessel clotting during the entire procedure (Video, Supplemental Digital Content 1); (II) the arteries were temporarily blocked, but the blood flow was almost completely restored within 5 min of injection (Fig. 3 and Video, Supplemental Digital Content 2); (III) the arteries were blocked and the main branch of central ear artery was not fully restored within 5 min of injection (Video, Supplemental Digital Content 3). There was no significant difference between the two PMMA groups, both of which had < 1/3 cases in category III, whereas 100% of the cases in both HA groups were in category III. The occurrence of category III was significantly higher in both HA groups than that in both PMMA groups (Fig. 4).

**Fig. 5:**
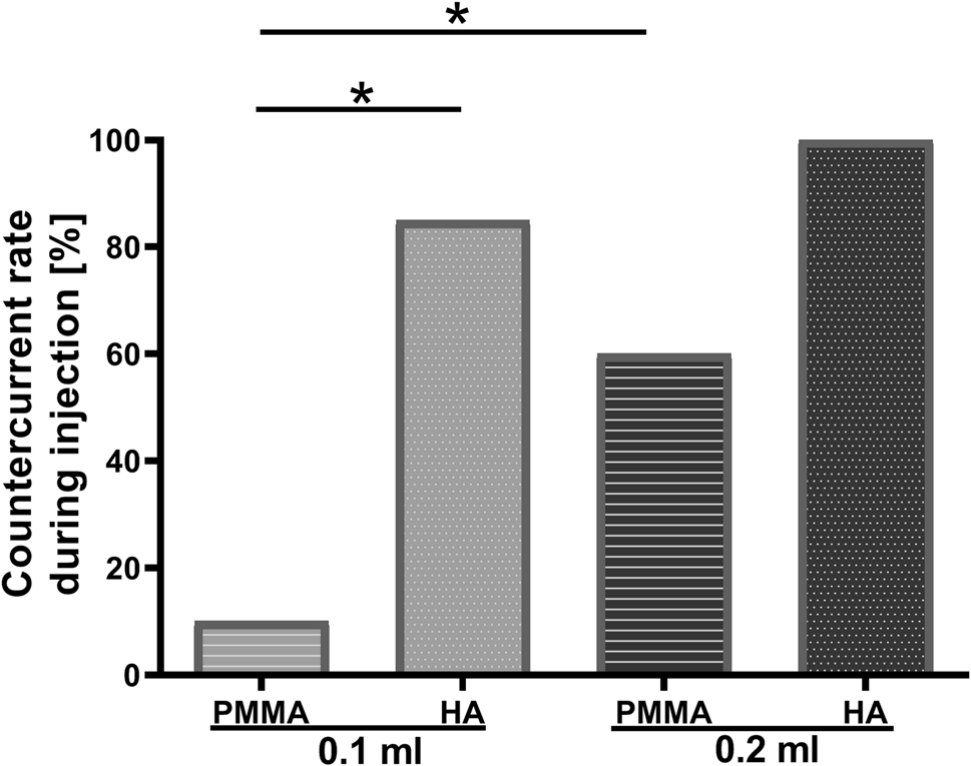
Countercurrent rate during injection. *p < 0.0125; *N* = 20 (0.1 ml groups) and 10 (0.2 ml groups)

In addition, the countercurrent rate in the 0.2 ml PMMA group (60%) was significantly higher than that in the 0.1 ml PMMA group (10%), indicating the effect of PMMA dose/volume on countercurrent (Fig. 5). At the same volume of 0.1 ml, HA treatment resulted in a higher countercurrent rate (85%) compared to that from PMMA treatment (10%), indicating that HA filler had a more dramatic effect on countercurrent (Fig. 5). Since the majority of HA-injected ears showed countercurrent (85% in 0.1 ml group and 100% in 0.2 ml HA group), no significant difference was observed between 0.1 ml HA and 0.2 ml HA groups (Fig. 5).

### Short-Term Effects of Intra-Arterial Injection of PMMA and HA on Vascular Occlusion and Skin Damage

**Fig. 6:**
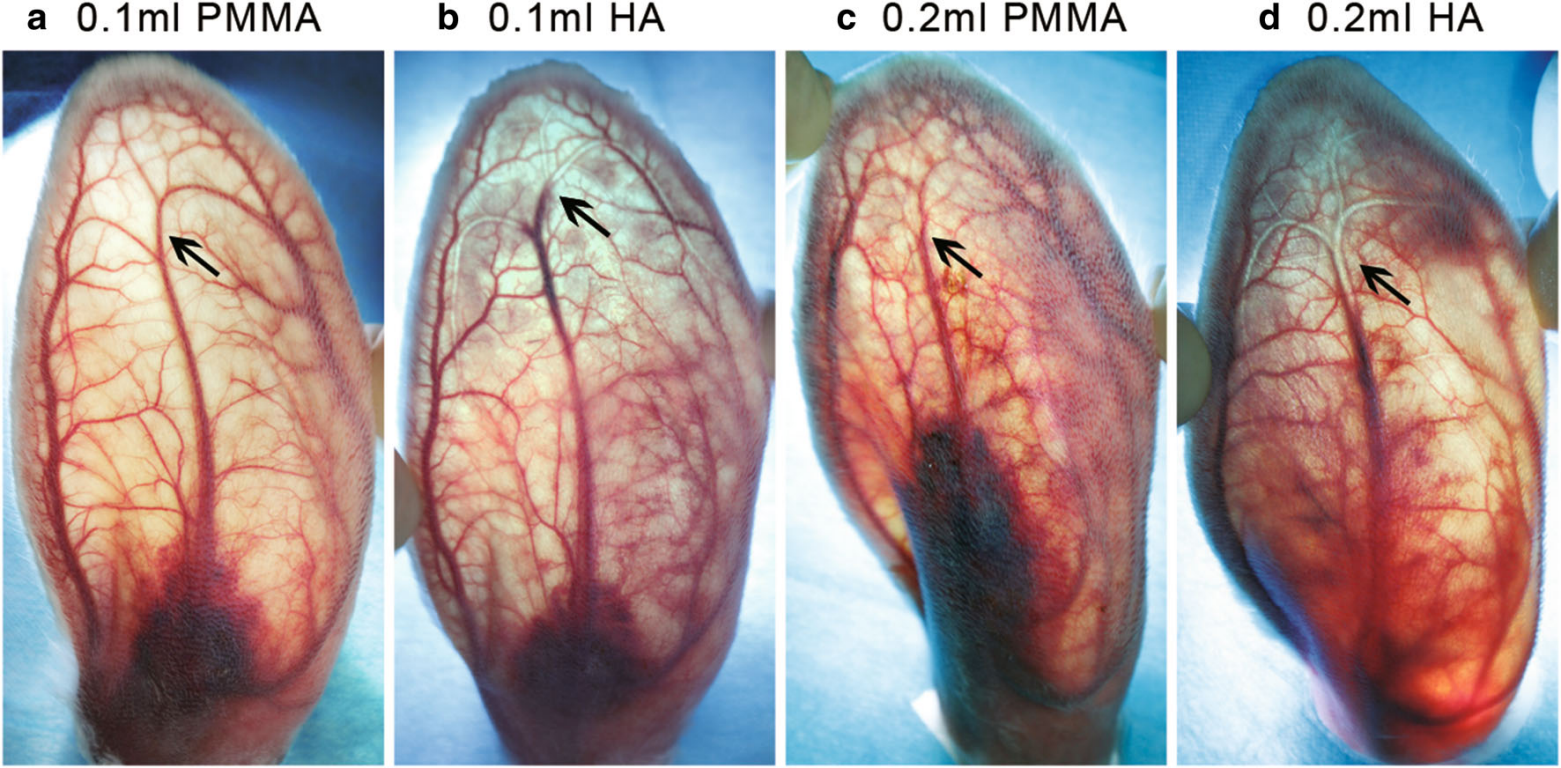
Representative images indicating blood flow perfusion of central ear artery (CEA) and its branches on day 1 after injection in each group. **a** 0.1 ml PMMA group. The blood flow was completely restored and the vascular texture was clear, only left an obvious hematoma at the injection site. **b** 0.1 ml HA group. The transparent embolus was limited to the terminal branches of CEA. **c** 0.2 ml PMMA group. There was no clear transparent embolus and no blockage of blood flow, but blurred vascular texture. **d** 0.2 ml HA group. There was transparent embolus in the branches of CEA and distal part of its main trunk. Arrows indicate the main point in each image

**Fig. 7:**
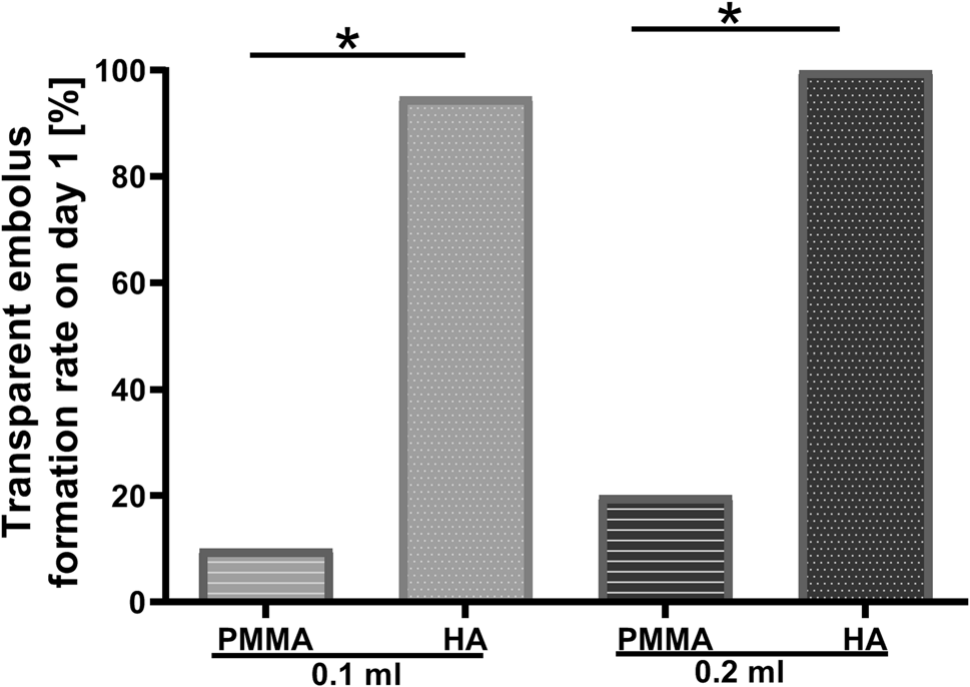
Percentage of visible transparent embolus detected on day 1 post-injection. *p < 0.0125; *N* = 20 (0.1 ml groups) and 10 (0.2 ml groups)

On day 1 post-injection, two phenomena were observed in the injected rabbit ears: without (Fig. 6a, c) or with (Fig. 6b, d) a transparent embolus, which can be seen in the branches of the central ear artery or even within its main trunk (Fig. 6b, d). Most of the ears (95% in 0.1 ml HA group and 100% in 0.2 ml HA group) injected with HA had a transparent embolus, whereas only 10–20% of PMMA-injected ears showed a transparent embolus, which was significantly lower than that in the HA-injected ears (Fig. 7). There was no significant difference between the two groups injected with 0.1 ml or 0.2 ml of the same fillers (PMMA or HA) (Fig. 7). These data demonstrated that HA was more effective than PMMA on the formation of a transparent embolus.

**Fig. 8:**
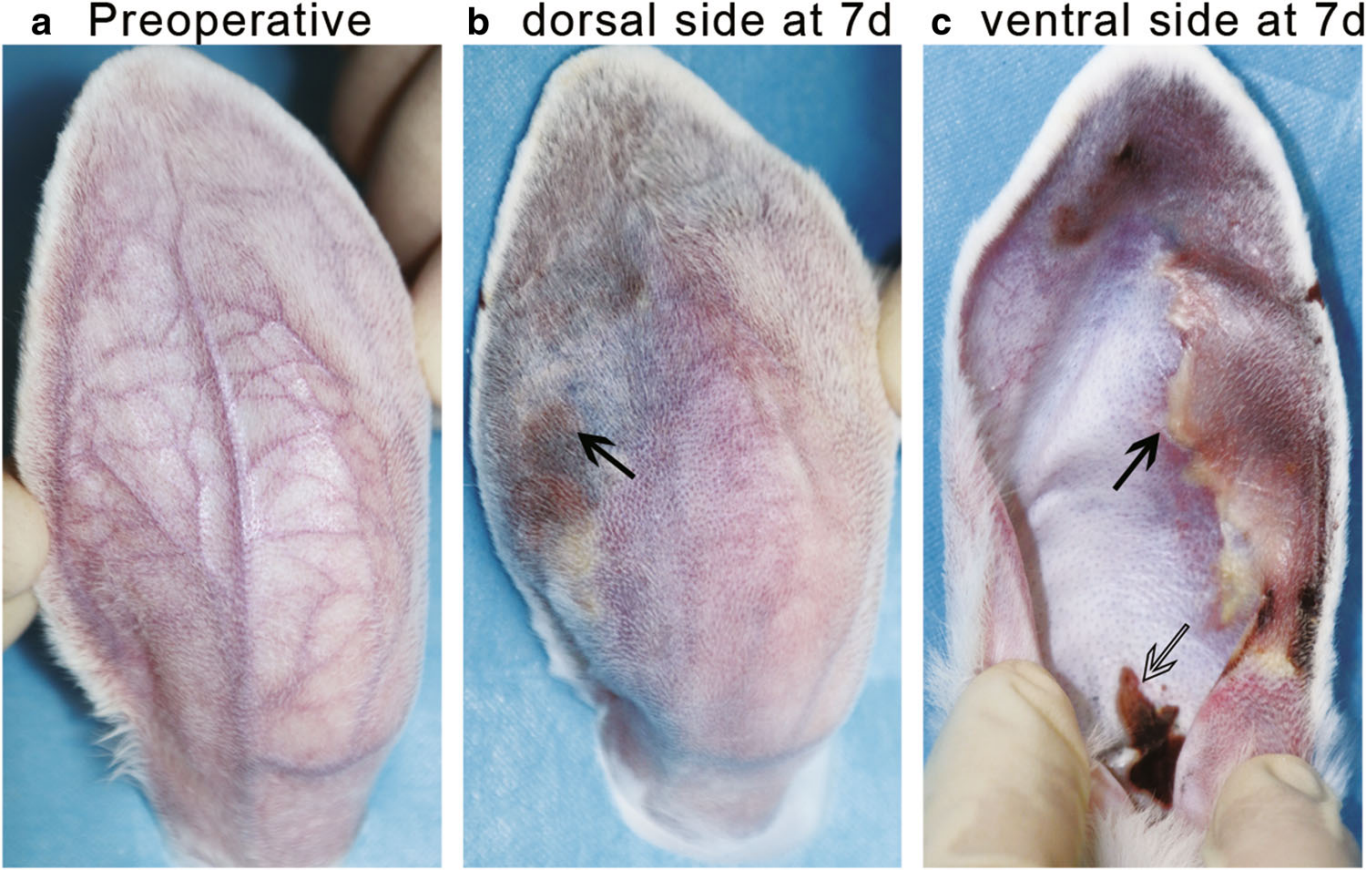
Appearances of an ear in the 0.2 ml HA group. **a** Before injection. **b** Dorsal side on day 7 post-injection. **c** Ventral side on day 7 post-injection. There was a large area of yellow and black skin bilaterally and an obvious destruction of the skin at the ear root of the ventral side, indicated by arrows

**Fig. 9:**
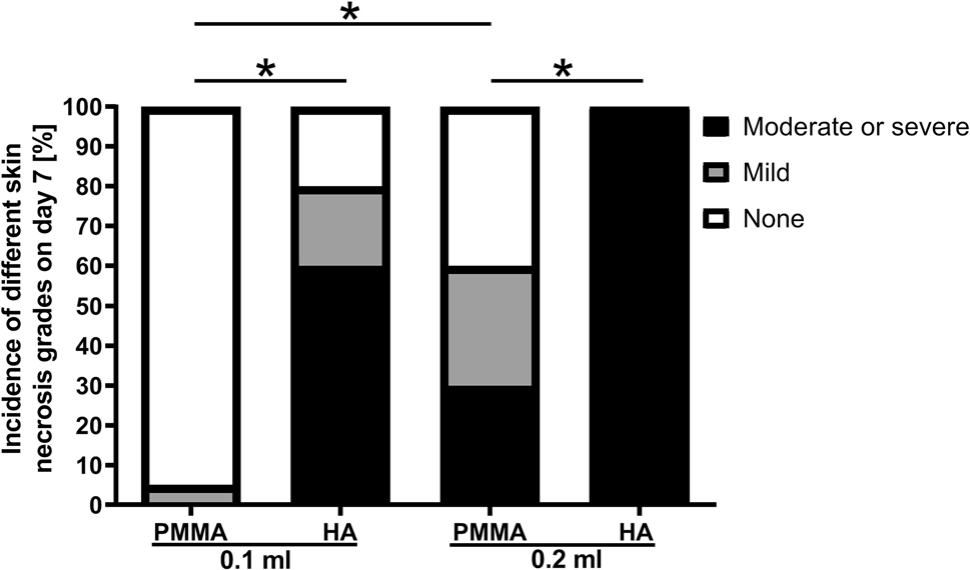
Incidence of skin necrosis on day 7 post-injection. White: no necrosis; gray: mild necrosis; black: moderate-to-severe necrosis. *p < 0.0125; *N* = 20 (0.1 ml groups) and 10 (0.2 ml groups)

On day 7 post-injection, the vascular occlusion status was similar or slightly recovered compared to that of day one (data not shown). However, different degrees of skin and soft tissue necrosis were observed in the injected ears on day 7: (1) none: The skin color and blood flow of the injected rabbit ears were comparable to that in the un-injected rabbit ears (Fig. 8a); (2) mild: The skin color on the ear was slightly darkened and/or the cartilage of the ear was slightly deformed; (3) moderate or severe (typical): The skin color turned yellow or black, and/or there was an obvious ulcer (Fig. 8b, c). At the same injection volumes (0.1 ml or 0.2 ml), HA had a more severe effect than PMMA on necrosis, with significantly higher percentages of HA-injected ears showing moderate or severe necrosis (Fig. 9). In addition, 4 out of 10 ears injected with 0.2 ml HA had skin ulcers at the ear root of the ventral side (Fig. 8c), which was not observed in the other three groups. In both PMMA- and HA-injected ears, 0.2 ml had a more severe effect than 0.1 ml on necrosis, especially in the PMMA-injected ears (Fig. 9), indicating that the injection volume was also a contributing factor to the severity of necrosis. Parallel to the occurrence of necrosis (Fig. 9), the area of necrosis was an additional indicator for the severity of the damaged skin and soft tissues (Table 1). The groups with higher incidences of skin and soft tissue damage also had larger areas of damaged skin and soft tissues (Fig. 9, Table 1).

**Table 1:** Areas of damaged skin and soft tissues on day 7 post-injection

Group	Median (cm^2^)	Min, max	Q25, Q75	^a^ *p*
A. 0.1 ml PMMA (*N* = 20)	0	0, 5.95	0, 0	0.000* (A vs. B)
B. 0.1 ml HA (*N* = 20)	5.58	0, 20.56	3.67, 7.96	0.007* (B vs. D)
C. 0.2 ml PMMA (*N* = 10)	1.16	0, 14.43	0, 9.87	0.026 (A vs. C)
D. 0.2 ml HA (*n* = 10)	17.28	9.53, 27.62	15.66, 18.86	0.001* (C vs. D)

Bold values are statistically significant (*p *< 0.0125)

^a^Nonparametric test * *p *< 0.0125

### Histopathological Changes of Blood Vessels on Day 7 Post-Injection

**Fig. 10:**
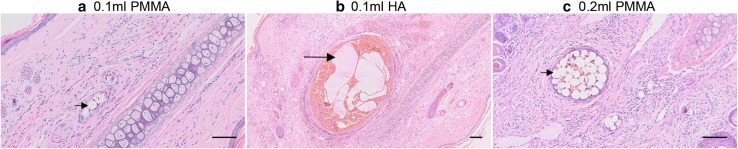
Histology of distal ear tissues on day 7 post-injection. **a** 0.1 ml PMMA group. There was a transparent and approximately 40 μm refractive microsphere (short arrowhead) in the deep dermis. No obvious abnormality was found in the tissue structure. **b** 0.1 ml HA group. The small blood vessel was dilated. The pink amorphous substance (long arrowhead) existed within a red thrombus. **c** 0.2 ml PMMA group. A cluster of microspheres (short arrowhead) was found in the deep dermis. Note: The magnification of the medium picture was a half of the other two. Scale bar, 100 μm

In the 0.1 ml PMMA group, the main histological change in the blood vessels was the presence of PMMA seen as transparent and refractive microspheres of about 40 μm in diameter (Fig. 10a). At most, a single PMMA microsphere was found in 7 out of 20 ear samples (35%) and more in the remaining 13 in this group. In the 0.2 ml PMMA group, the main histological change was similar to the 0.1 ml PMMA group except that more microspheres were detected in the branches of central ear artery and microvessels (Fig. 10c). Interestingly, PMMA microspheres were also detected in the main trunk of the central ear artery in 2 out of 10 ears in this group.

In the 0.1 ml HA group, the histological changes of the blood vessels were mainly in the distal segment of the ears. The small vessels or microvessels were dilated, and pink or lilac amorphous substances (HA) were found in the lumen (Fig. 10b). In the 0.2 ml HA group, in addition to similar changes in the distal segment, dilated vessels and HA in the lumen can be seen in the main trunks of the central ear artery in 8 out of 10 cases.

The diameters of the largest vessels were 384.24 ± 130.80 μm (0.1 ml PMMA), 951.89 ± 366.09 μm (0.1 ml HA), 632.44 ± 258.32 μm (0.2 ml PMMA), and 848.14 ± 202.47 μm (0.2 ml HA), respectively. There was a significant difference between the 0.1 ml PMMA group and the 0.1 ml HA group (Table 2).

**Table 2:** Diameters of the largest vessels on day 7 post-injection

Group	Mean (µm)	SD	SE mean	^a^ *p*
A. 0.1 ml PMMA (*N* = 20)	384.24	130.80	29.25	**0.000 (A vs. B)**
B. 0.1 ml HA (*N* = 20)	951.89	366.09	81.86	0.717 (B vs. D)
C. 0.2 ml PMMA (*N* = 10)	632.44	258.32	81.69	0.024 (A vs. C)
D. 0.2 ml HA (*n* = 10)	848.14	202.47	64.03	0.129 (C vs. D)

Bold value is statistically significant (*p *< 0.0125)

^a^ Nonparametric test * *p *< 0.0125

## Discussion

Arterial occlusion is considered to be the most dangerous and aggressive complication from application of fillers. There are few methods available to treat this complication. Most experts agree that anatomical knowledge and injection technology are important to prevent vascular complications [2]. As far as we know, there is no animal experimental evidence that different fillers have different risks of intravascular embolism.

Based on the latest literature, reports of HA-induced vascular embolism are far more than those of PMMA [1, 9]. There have been several explanations for this phenomenon: (1) the injection planes recommended for Artecoll (PMMA) include the periosteum surface or the dermal–subdermal plane [7, 10, 11], whereas Restylane (HA) is intended to be used for facial tissue augmentation by injections into the mid-dermis; (2) PMMA is not as popular as HA [2], and thus, less adverse events were reported for PMMA; (3) HA has a large particle size, increased hydrophilicity, and viscosity, and thus, it could result in more proximal vessel obstructions [1, 5]. To verify the correlation between the physical properties of the fillers and the arterial embolization risks, the rabbit ear was used as a model in this study.

In the rabbit ears, the skin is thin and the blood vessel distribution is shallow; it is easy to observe vascular embolisms. During the injection, Artecoll was easily disbanded with blood flow, and it was not easy to reflux to the proximal vessels compared with HA at 0.1 ml. Also, obvious blood leakage in the injection site was seen within seconds of needle removal. These phenomena confirmed that PMMA does not tend to completely block blood vessels because of its low viscosity and fine granule constituents. The rate of filler embolus formation in HA groups on day 1 was significantly higher than that of Artecoll groups with the same injection volumes. Correspondingly, HA injection led to obvious skin tissue necrosis on day 7 post-injection. These results support our hypothesis that different fillers have different risks of vascular embolization and corroborate higher incidences of vascular complications associated with HA application [4].

On the other hand, reports on tissue necrosis or ophthalmic artery embolization caused by soft tissue filler containing PMMA components are relatively rare, but it could be serious once it occurs [9, 12–14]. Although the accurate dose of the injection was not mentioned in these reports, we speculate that it may be related to larger volumes or fast injection into the blood vessels. This speculation was supported by the observations that more damage was observed in the 0.2 ml PMMA group than in the 0.1 ml PMMA group. It indicates that the intravascular injection volume plays an important role in the outcomes. Similarly, 0.2 ml HA resulted in necrosis in all cases, whereas 0.1 ml HA had the possibility of skin survival in some cases. Individual differences may include vascular diameters, collateral circulation, hemodynamics, etc. The existing clinical data [1] show that in some cases no skin necrosis appeared although fillers were found in the artery after injection, which may be associated with lower injection doses and individual differences in patients.

In the 0.2 ml HA group, necrosis occurred in all the injected ears. Dorsal skin necrosis of the ear root was observed in some cases, which might be caused by reverse flow of HA that entered the artery branch of the dorsal skin. Based on our study, reverse flow was related to the materials and injection volumes. It is possible that HA at an injection volume over 0.2 ml to the central ear artery may cause a more serious complication such as intracranial arterial embolization. This is similar to the ophthalmic artery embolization in clinical cases [15].

Histological examination indicated that compared with Artecoll, HA caused an obvious expansion of the lumen of the embolic vessels, resulting in a more serious blockage of blood supply. According to instructions from the manufacturer, the particle sizes (measured as swelled) of 80% Restylane 2 range from 80 to 1000 μm. Although the diameter of a single PMMA microsphere is about 40 μm, a large cluster of PMMA microspheres may also block a blood vessel. Therefore, large doses of PMMA could also cause complete blockage of blood vessels. These observations suggest that avoiding single injections of large doses of fillers at a single site could help reduce the complications of vascular embolism.

In summary, we demonstrated the risk differences between different fillers (PMMA and HA) and different doses (0.1 ml and 0.2 ml) on vascular complications. To reduce the vascular complications from injections of fillers, we need to pay attention to the following three aspects: (1) the selection of an appropriate filler for injection; (2) the selection of appropriate site and tissue plane for injection; and (3) the use of small volumes and the control of speed for injection. Further studies are needed for optimal applications of different fillers.

## Conclusion

Under our experimental setting, HA causes more severe complications that PMMA does, which supports our clinical observations. Our study indicates that both the nature of the filler and the injection volume could contribute to the outcomes of filler applications.

## Electronic Supplementary Material

Below is the link to the electronic supplementary material.

Video, Supplemental Digital Content 1. Rapid dispersion of PMMA in blood flow without blockage during the whole procedure (one case in 0.1 ml PMMA group). (MP4 38853 kb)

Video, Supplemental Digital Content 2. Restoration of blood flow after temporary blockage within 5 min after injection. (MP4 39687 kb)

Video, Supplemental Digital Content 3. Complete blockage of blood flow within 5 min after injection. (MP4 39843 kb)
